# What choanoflagellates can teach us about symbiosis

**DOI:** 10.1371/journal.pbio.3002561

**Published:** 2024-04-03

**Authors:** Arielle Woznica

**Affiliations:** Department of Molecular Biosciences, The University of Texas at Austin, Austin, Texas, United States of America; University of Oxford, UNITED KINGDOM

## Abstract

Environmental bacteria influence many facets of choanoflagellate biology, yet surprisingly few examples of symbioses exist. This Perspective explores why choanoflagellates are an exciting system for enriching our understanding of eukaryote-bacteria symbioses and for considering how symbiotic associations with bacteria may shaped the early evolution of animals.

## Teaser

Environmental bacteria influence many facets of choanoflagellate biology, yet surprisingly few examples of symbioses exist. We need to find out why, as choanoflagellates can help us to understand how symbiosis may have shaped the early evolution of animals.

Long before the first animals appeared, their protozoan ancestors had already established crucial relationships with bacteria that likely ranged from predation to symbiosis. Animal model systems have yielded insights into the diverse biological functions of, and molecular rules governing, animal–bacteria symbioses today. Yet, we still know relatively little about the evolutionary foundations of these associations. The closest unicellular relatives of animals, the choanoflagellates, are uniquely positioned to reveal how symbiotic interactions with bacteria may have shaped the biology of animal progenitors and influenced animal origins.

**Fig 1:**
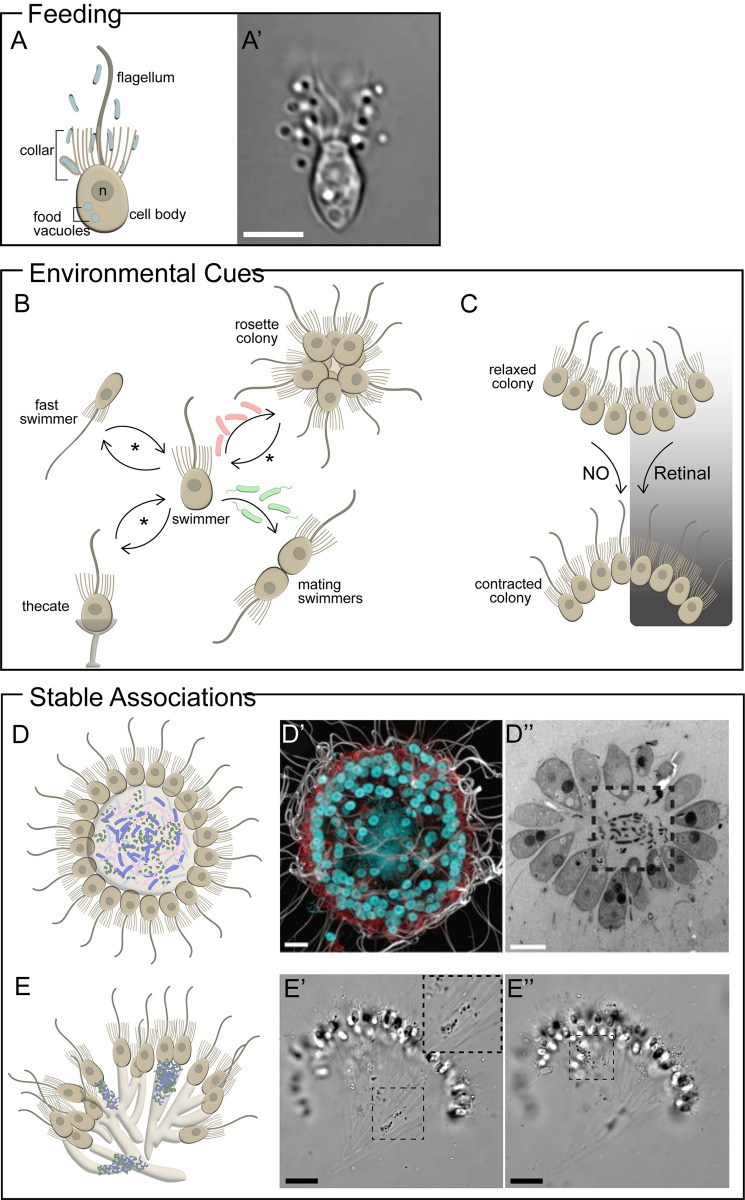
Choanoflagellate–bacteria interactions. **(A)** Choanoflagellates survive by eating bacteria. Schematic of a feeding choanoflagellate cell highlighting their “collar complex”: the apical flagellum surrounded by an actin-filled microvilli collar (A). Flagellar beating draws bacterial prey (blue) into the collar, where they become trapped and phagocytosed at the collar membrane. Bacteria are later digested in food vacuoles. n = nucleus. DIC image of *Salpingoeca rosetta* consuming environmental bacteria (A′). Scale bar = 5 μm. **(B)** Bacterial cues regulate *S*. *rosetta* developmental transitions. Specific cues produced by environmental bacteria regulate rosette development and sexual reproduction. Lipid cofactors produced by *Algoriphagus machipongonensis* act synergistically to regulate multicellular rosette development in unicellular swimmer cells. A chondroitin lyase produced by *Allivibrio fischeri* induces swimmer cells to mate and undergo sexual reproduction. Other *S*. *rosetta* developmental transitions are influenced by nutrient availability (*), and we hypothesize that these might also be regulated by bacteria. **(C)** Common bacterially produced metabolites induce collective cell contractions in *Choanoeca flexa*. Cell contractions that result in colony inversion can be triggered either by exogenous nitric oxide (NO) or by light-to-dark transitions in the presence of retinal produced by environmental bacteria. **(D)** *Barroeca monosierra* forms stable, physical associations with bacteria (D). Maximum intensity projection of an immunostained *B*. *monosierra* colony shows that the hollow center is filled with bacterial DNA, revealed by Hoechst staining (D′). Apical flagella are highlighted in white, microvilli are highlighted in red, and nuclei are highlighted in cyan. Thin section through an *S*. *monosierra* colony, imaged by transmission electron microscopy, reveals the presence of bacteria in the central cavity (D′′). Figure adapted from [11]. Scale bars = 5 μm. **(E)** *Choanoeca* sp. produce tubes of extracellular matrix that are stably colonized by bacteria (E). DIC imaging of a single *Choanoeca* sp. colony at 2 different Z positions shows bacteria colonizing the interior (E′) and surface (E′′) of tubed projections. Scale bars = 20 μm.

Choanoflagellates, the sister group to animals, are microeukaryotes that live in freshwater and marine environments around the world, where they exist in diverse single-celled and colonial morphologies [1]. As predators of bacteria, choanoflagellates use their signature “collar complex” (Fig 1A), consisting of a single apical flagellum surrounded by a microvilli feeding collar, to capture environmental bacteria for phagocytosis (notably, the role of the collar complex in mediating interactions with bacteria is exclusively conserved among choanoflagellates and animals [2]). However, bacteria are not just food! Choanoflagellates initiate a range of developmental transitions and collective behaviors in response to environmental bacteria and can also form stable associations with bacterial communities. While it is evident that bacteria influence many core features of choanoflagellate biology, we have observed surprisingly few examples of stable, physical associations between choanoflagellates and bacteria. More often, we find that short-lived interactions with environmental bacteria are sufficient to regulate choanoflagellate cellular responses (at least under laboratory conditions). This leaves us asking what symbiosis means to choanoflagellates. Have choanoflagellates evolved to rely on transient interactions with bacteria in lieu of forming physical associations? Is it possible for seemingly transient relationships with environmental bacteria to be symbiotic? Or maybe stable, physical partnerships between choanoflagellates and bacteria are more widespread than we realize, but just challenging to identify.

Although the feeding behavior of choanoflagellates has fascinated microscopists since the mid-nineteenth century, environmental bacteria were not observed to regulate choanoflagellate biology until 150 years later, when Nicole King’s group began studying the choanoflagellate *Salpingoeca rosetta*. As is true for many choanoflagellate species, *S*. *rosetta* can develop from a single cell into a multicellular colony through serial rounds of oriented cell divisions [3]. Although *S*. *rosetta* was initially isolated from the environment as a multicellular “rosette” colony (Fig 1B), rosettes quickly transitioned to single-celled states when cultured in the lab. A series of serendipitous experiments revealed that the co-isolated environmental bacterium, *Algoriphagus machipongonensis*, induces single cells to develop into rosette colonies [4]. Later experiments led to the unexpected finding that bacteria regulate another, very different developmental decision in *S*. *rosetta*: some species of *Allivibrio* and *Vibrio* bacteria, including *Allivibrio fischeri*, induce the switch to sexual reproduction (Fig 1B) [5].

Bacteria are reliable proxies for environmental conditions, and *S*. *rosetta* is among a contingent of diverse eukaryotes that make important decisions in response to environmental bacteria; for example, external bacteria also stimulate algal differentiation and zoospore settlement, and bacteria-induced metamorphosis is widespread among animals [6]. Despite sharing the feature of transience, relationships between eukaryotes and their environmental bacteria can have vastly different evolutionary histories and specificities. The choanoflagellate *Choanoeca flexa* can use nitric oxide and retinal, both metabolites produced by diverse bacteria, to initiate collective cell contractions [7,8] (Fig 1C). Cell contractions in *C*. *flexa* toggle colonies between morphologies that favor either feeding or swimming; because cells have evolved responses to such common bacterial metabolites, *C*. *flexa* is able to use bacteria to navigate diverse environments.

By contrast, the interaction between *S*. *rosetta* and rosette-inducing *Algoriphagus* bacteria is remarkably specific. *Algoriphagus* produces distinct classes of lipid co-factors that act synergistically to regulate rosette development [4,9]. The molecular stringency required for rosette development (which is warranted, seeing as the transition to multicellularity is a serious commitment) raises the possibility that populations of *S*. *rosetta* and *Algoriphagus* have lived in close association over time. Could this be a form of symbiosis? Much of what we know about symbiosis comes from obligately multicellular animal hosts. Yet, the life histories of animals and choanoflagellates are different: choanoflagellates have short generation times and unicellular life-stages. Perhaps some choanoflagellate, and possibly other microbial, symbioses have evolved to manifest at the population level (across environmental space) rather than the individual level. This way, choanoflagellates can rely on specific relationships with bacteria while maintaining the ability to nimbly respond to environmental fluctuations.

Exploring the idea that transient associations with bacteria can verge on symbiotic requires us to study choanoflagellates both in the lab and in the wild. While laboratory studies provide critical information about the molecular underpinnings of choanoflagellate–bacteria interactions (which can help us hypothesize about coevolution), they cannot offer much ecological context. Because choanoflagellates are small and live in fluctuating environments, consistently capturing choanoflagellate–bacteria interactions using classic microscopy-based isolation techniques has proven challenging. Thus, we need to incorporate new approaches to understand how prevalent or stable specific associations are in nature. For instance, metagenomic sequencing and cell sorting methods that enrich for choanoflagellates will be key for sampling numerous microenvironments to track associations over space and time. Similar methods may also prove valuable for identifying new symbioses between choanoflagellates and bacteria. Single-cell sorting and sequencing choanoflagellates in the field has already revealed a co-association between the uncultivated choanoflagellate *Bicosta minor* and a previously uncharacterized bacterium [10]. Interestingly, the co-isolated bacterium has a reduced genome that is suggestive of a host-dependent lifestyle. Yet, because this association is based solely on genomic data and has not been visualized, the details of this interaction remain ambiguous. Nonetheless, similar culture-independent approaches have enormous potential to help us uncover ecologically relevant choanoflagellate–bacteria interactions and symbioses.

If forming associations with environmental bacteria enables choanoflagellates to navigate diverse environmental contexts, what might prompt choanoflagellates to establish stable, physical symbioses with bacteria? And are these symbioses restricted to specific life-history stages? The species *Barroeca monosierra* provides a visually striking example of choanoflagellate symbiosis, forming large and spherical colonies that stably associate with bacteria [11] (Fig 1D). As *B*. *monosierra* colonies grow, environmental bacteria can colonize their hollow centers to establish a microbial community comprised of several coexisting species. The drivers and functions of this symbiosis are still unknown, but it does not hurt to speculate. Choanoflagellates both acquire essential nutrients from eating bacteria and harbor many amino acid biosynthesis pathways that were lost in animals, so it is unlikely that *B*. *monosierra* depends on its microbiome solely for nutritional supplementation. Yet, because the natural habitat of *B*. *monosierra* is a hypersaline, alkaline lake, it seems plausible that these interactions are based on metabolism or detoxification. In turn, these associations may be driven by bacteria, and the extracellular matrix of large *B*. *monosierra* colonies may serve as a nutrient-rich niche for environmental bacteria to exploit. Our recent observation that bacteria also stably colonize the extracellular matrix of a newly identified *Choanoeca* sp. suggests that similar choanoflagellate–bacteria symbioses may be more prevalent than we realize. Cells within a *Choanoeca* sp. colony are connected by branched tubes of extracellular matrix, resulting in a tree-like appearance (Fig 1E, AW unpublished results). Symbiotic bacteria colonize discrete patches on the surface and within the center of these hollow tubes, although it remains unclear if the bacteria belong to one or more species. The natural habitat of *Choanoeca* sp. (isolated from a tropical tide pool) differs from that of *B*. *monosierra*, yet it is possible that forming ectosymbioses enables both species to withstand environmental stresses. As we being to explore how and why choanoflagellates establish physical symbioses, it will be important to study these interactions in the lab under a range of conditions, and ideally, in nature.

Although interactions with environmental bacteria influence most facets of choanoflagellate biology, we have currently gained but a glimpse into choanoflagellate–bacteria symbioses. Nearly every lineage of eukaryotes, from multicellular animals to unicellular protists, forms different symbiotic associations with bacteria. So why have choanoflagellate symbioses remained so elusive? This can be explained in part by cultivation bias, and in part by human bias (we were not looking). In addition, we have likely failed to recognize known interactions as symbiotic because they have characteristics that are tricky to categorize. While some associations with bacteria clearly resemble animal symbioses, others blur the line between transient and symbiotic depending on context. Nonetheless, pursuing studies of choanoflagellate symbiosis is well worth the challenge: These unique organisms have the potential to enrich our understanding of microbial symbioses while providing exceptional insights into the fundamental mechanisms and evolutionary history of animal–bacteria associations.

## References

[1] Leadbeater BSC. The Choanoflagellates: Evolution, Biology and Ecology. Cambridge University Press; 2015. p. 18–43. doi: 10.1017/cbo9781139051125.003

[2] Brunet T, King N. The Origin of Animal Multicellularity and Cell Differentiation. Dev Cell. 2017;43(124):140. doi: 10.1016/j.devcel.2017.09.016 29065305 PMC6089241

[3] Fairclough SR, Dayel MJ, King N. Multicellular development in a choanoflagellate. Curr Biol. 2010;20:R875–R876. doi: 10.1016/j.cub.2010.09.014 20971426 PMC2978077

[4] Alegado RA, Brown LW, Cao S, Dermenjian RK, Zuzow R, Fairclough SR, et al. A bacterial sulfonolipid triggers multicellular development in the closest living relatives of animals. elife. 2012;1:e00013. doi: 10.7554/eLife.00013 23066504 PMC3463246

[5] Woznica A, Gerdt JP, Hulett RE, Clardy J, King N. Mating in the Closest Living Relatives of Animals Is Induced by a Bacterial Chondroitinase. Cell. 2017;170(1175):1183.e11. doi: 10.1016/j.cell.2017.08.005 28867285 PMC5599222

[6] Woznica A, King N. Lessons from simple marine models on the bacterial regulation of eukaryotic development. Curr Opin Microbiol. 2018;43:108–116. doi: 10.1016/j.mib.2017.12.013 29331767 PMC6051772

[7] Brunet T, Larson BT, Linden TA, Vermeij MJA, McDonald K, King N. Light-regulated collective contractility in a multicellular choanoflagellate. Science. 2019;366:326–334. doi: 10.1126/science.aay2346 31624206

[8] Reyes-Rivera J, Wu Y, Guthrie BGH, Marletta MA, King N, Brunet T. Nitric oxide signaling controls collective contractions in a colonial choanoflagellate. Curr Biol. 2022;32:2539–2547.e5. doi: 10.1016/j.cub.2022.04.017 35504284

[9] Woznica A, Cantley AM, Beemelmanns C, Freinkman E, Clardy J, King N. Bacterial lipids activate, synergize, and inhibit a developmental switch in choanoflagellates. Proc Natl Acad Sci U S A. 2016;113:7894–7899. doi: 10.1073/pnas.1605015113 27354530 PMC4948368

[10] Needham DM, Poirier C, Bachy C, George EE, Wilken S, Yung CCM, et al. The microbiome of a bacterivorous marine choanoflagellate contains a resource-demanding obligate bacterial associate. Nat Microbiol. 2022;7:1466–1479. doi: 10.1038/s41564-022-01174-0 35970961 PMC9418006

[11] Hake K, West PT, McDonald KL, Laundon D, Feng C, Burkhardt P, et al. Colonial choanoflagellate isolated from Mono Lake harbors a microbiome [preprint]. bioRxiv. 2021 Nov 10 [cited 2023 Feb 16]. doi: 10.1101/2021.03.30.437421

